# The Ehrlich Tumor Induces Pain-Like Behavior in Mice: A Novel Model of Cancer Pain for Pathophysiological Studies and Pharmacological Screening

**DOI:** 10.1155/2013/624815

**Published:** 2013-08-29

**Authors:** Cassia Calixto-Campos, Ana C. Zarpelon, Mab Corrêa, Renato D. R. Cardoso, Felipe A. Pinho-Ribeiro, Rubens Cecchini, Estefania G. Moreira, Jefferson Crespigio, Catia C. F. Bernardy, Rubia Casagrande, Waldiceu A. Verri

**Affiliations:** ^1^Departamento de Ciências Patológicas, Centro de Ciências Biológicas, Universidade Estadual de Londrina, Rod. Celso Garcia Cid KM480 PR445, 86051-990 Londrina, PR, Brazil; ^2^Departamento de Ciências Fisiológicas, Centro de Ciências Biológicas, Universidade Estadual de Londrina, Rod. Celso Garcia Cid KM480 PR445, 86051-990 Londrina, PR, Brazil; ^3^Departamento de Enfermagem, Centro de Ciências da Saúde, Universidade Estadual de Londrina, Avenida Robert Koch 60, 86038-350 Londrina, PR, Brazil; ^4^Departamento Ciências Farmacêuticas, Centro de Ciências da Saúde, Universidade Estadual de Londrina, Avenida Robert Koch 60, 86038-350 Londrina, PR, Brazil

## Abstract

The Ehrlich tumor is a mammary adenocarcinoma of mice that can be developed in solid and ascitic forms depending on its administration in tissues or cavities, respectively. The present study investigates whether the subcutaneous plantar administration of the Ehrlich tumor cells induces pain-like behavior and initial pharmacological susceptibility characteristics. The Ehrlich tumor cells (1 × 10^4^–10^7^ cells) induced dose-dependent mechanical hyperalgesia (electronic version of the von Frey filaments), paw edema/tumor growth (caliper), and flinches compared with the saline group between days 2 and 12. There was no difference between doses of cells regarding thermal hyperalgesia in the hot-plate test. Indomethacin (a cyclooxygenase inhibitor) and amitriptyline hydrochloride (a tricyclic antidepressant) treatments did not affect flinches or thermal and mechanical hyperalgesia. On the other hand, morphine (an opioid) inhibited the flinch behavior and the thermal and mechanical hyperalgesia. These effects of morphine on pain-like behavior were prevented by naloxone (an opioid receptor antagonist) treatment. None of the treatments affected paw edema/tumor growth. The results showed that, in addition to tumor growth, administration of the Ehrlich tumor cells may represent a novel model for the study of cancer pain, specially the pain that is susceptible to treatment with opioids, but not to cyclooxygenase inhibitor or to tricyclic antidepressant.

## 1. Introduction 

Pain is a symptom related to poor quality of life in cancer patients. In fact, in the United States, it is the most frequent cause of disability in these patients [1, 2]. Furthermore, reports of cancer pain have been increasing over the years accompanying the increased survival of patients [3, 4]. Most patients with advanced cancer (60%–85%) and 5-year survivors (40%) report pain [5–8]. In patients with advanced cancer, 62%–85% experience significant pain that is described as moderate to severe in approximately 4%–50% and as very severe in 25%–30% [9]. In fact, approximately 43% of the patients report feeling pain as early as diagnosis [7]. Therefore, pain management in cancer patients is a public health issue, and the mechanisms of cancer pain are not completely understood [7]. 

In this sense, there are various animal models of cancer pain that are used in an attempt to clarify the nociceptive pathways involved in cancer-related pain, including skin cancer pain [10], neuropathic cancer pain [11], and bone cancer pain [12, 13]. These models have been important, for instance, in the demonstration of the contribution of transient receptor potential vanilloid receptor 1 (TRPV1), acid-sensing ion channels (ASICs), nerve growth factor (NGF), bradykinin, adenosine triphosphate (ATP), endothelin, and other mediators in the nociceptor sensitization during cancer pain [14]. There is also evidence that the inflammatory response against the tumor cells results in the production of cytokines and chemokines that sensitize the nociceptors by receptor-mediated activation of protein kinase C (PKC) and protein kinase A (PKA) and/or activation of mytogen-activated protein kinases such as p38. The activation of these intracellular pathways results in activation of TRPV1 and tetrodotoxin-resistant sodium channels and increased expression of TRPV1 [15]. Therefore, there are complex mechanisms, which may also vary depending on the cancer type.

The Ehrlich tumor is a spontaneous murine mammary adenocarcinoma [16] adapted to ascites form [17] and carried in mice by serial intraperitoneal (i.p.) passages [18]. The ascitic form of the tumor has been used as experimental model to assess the influence of drugs of different origins on its proliferation and host responses against the tumor cells [19–21]. The characteristic ascites is probably formed as a consequence of the inflammatory response towards tumor cells resulting in increased peritoneal vascular permeability [22]. Other factors that contribute to ascites and lethality of the Ehrlich tumor includes the impaired peritoneal lymphatic drainage by the tumor cells [22], the mechanic pressure exerted by progressive increase of ascitic fluid, peritoneal hemorrhage, and endotoxemia [23–25]. The Ehrlich tumor cells are also used as a model of solid tumor by injection in different sites [26]. 

Despite the wide use of the Ehrlich tumor cells in the investigation of the mechanisms of tumor proliferation as well as the host inflammatory and oxidative responses against tumor cells, it is yet undetermined whether inoculation of the Ehrlich tumor cells could represent a murine model to study cancer pain. Therefore, the present study standardized a murine model of cancer pain induced by the intraplantar injection of the Ehrlich tumor cells and investigated the pharmacological susceptibility of the model using three classes of analgesics. 

## 2. Materials and Methods

### 2.1. Animals

The experiments were performed on male Swiss mice (20–25 g, Universidade Estadual de Londrina, Londrina, PR, Brazil) housed in standard clear plastic cages (six per cage) with free access to food and water. The behavioral testing was performed between 9:00 am and 5:00 pm in a temperature-controlled room. Animals' care and handling procedures were in accordance with the International Association for Study of Pain (IASP) guidelines and with the approval of the Ethics Committee of Universidade Estadual de Londrina. All efforts were made to minimize the number of animals used and their suffering. 

### 2.2. The Ehrlich Tumor Cells Inoculation

The Ehrlich tumor cells were collected from ascitic fluid of the peritoneal cavity of mice 10 days after tumor administration. The ascitic fluid was washed in phosphate-buffered saline (PBS, pH 7.4), centrifuged (200 g, 10 min), and washed with PBS three times. The cell viability was determined by the 0.5% trypan blue exclusion method in the Neubauer chamber [27]. The Ehrlich tumor cells were suspended to the final concentrations of 1 × 10^4^, 1 × 10^5^, 1 × 10^6^, and 1 × 10^7^ in 25 *μ*L of saline. Measurements were performed before and after injection of tumor cells between days 0 and 12.

### 2.3. Drugs

Drugs were obtained from the following sources: indomethacin from Prodome Chemical and Pharmaceutical (Sao Paulo, SP, Brazil), amitriptyline from Germed (Sao Bernardo do Campo, SP, Brazil), morphine sulphate from Cristalia (São Paulo, SP, Brazil), and naloxone hydrochloride from Sigma-Aldrich (St Louis, MO, USA).

### 2.4. Protocols

Firstly, mice received intraplantar (i.pl.) injection of the Ehrlich tumor cells (1 × 10^4^–10^7^ in 25 *μ*L) or saline. Measurements of mechanical and thermal hyperalgesia, paw edema/tumor growth, and overt pain-like behavior were performed on days 0–12. According to the results, the dose of 1 × 10^6^/paw of tumor cells was chosen for next experiments of mechanical hyperalgesia, thermal hyperalgesia, paw edema/tumor growth, and histological analysis at indicated timepoints. The dose of 1 × 10^7^/paw of tumor cells and evaluation at the 8th day after inoculation were chosen for experiments of overt pain. Paw samples were collected for histological analysis and microscopic observation 12 days after tumor injection. To evaluate the hyperalgesic effect of cellular remnants, the Ehrlich tumor cells were inactivated and injected i.pl., and compared with the saline and the viable Ehrlich tumor cells groups; measurements were performed on days 0–12. To evaluate the pharmacological modulation of the Ehrlich tumor-induced pain-like behavior, mice were treated with the cyclooxygenase inhibitor (indomethacin, 0.7, 2, and 6 mg/kg, i.p.) or opioid (morphine, 1, 3, and 10 mg/kg, i.p.) on the 8th day after the Ehrlich tumor cells administration, and the evaluation of mechanical and thermal hyperalgesia, paw edema/tumor growth, and overt pain was performed 3 h or 45 min after the treatment, respectively. Another group of mice was treated with tricyclic antidepressant (amitriptyline, 3, 10, and 30 mg/kg, p.o.) daily during 12 days. The evaluation of mechanical and thermal hyperalgesia, paw edema/tumor growth, and overt pain was performed 3 h after treatment on days 0–12. It is noteworthy that different experimenters prepared the solutions, made the administrations, and performed the evaluation of pain-like behavior.

### 2.5. The Electronic Pressure Meter Test of Mechanical Hyperalgesia

Mechanical hyperalgesia was tested in mice as previously reported [28]. Briefly, the test consists of evoking a hindpaw flexion reflex with a hand-held force transducer (the electronic von Frey anesthesiometer: Insight, Ribeirão Preto, SP, Brazil) adapted with a 0.5 mm^2^ contact area polypropylene tip. The investigator was trained to apply the tip perpendicularly to the central area of the hindpaw, and the endpoint was characterized by the removal of the paw. The results are expressed by delta (Δ) withdrawal threshold (in g), which was calculated by subtracting the zero-time mean measurements from the mean measurements (indicated timepoints) after stimulus.

### 2.6. The Hot-Plate Test of Thermal Hyperalgesia

Thermal hyperalgesia was evaluated before and at indicated timepoints after injection of the Ehrlich tumor cells. In brief, mice were placed in a 10 cm wide glass cylinder on a hot plate (Hot Plate HP-2002, Insight Equipamentos, Ribeirao Preto, SP, Brazil) maintained at 55°C. The reaction time was scored when the animal jumped, flinched, and/or licked its paws. A maximum latency (cutoff) was set at 30 s to avoid tissue damage [29].

### 2.7. Evaluation of Paw Edema/Tumor Growth

The paw edema/tumor growth was determined before and at indicated timepoints (at 48 h intervals) after the injection of the Ehrlich tumor cells using an analog caliper. Paw edema/tumor growth was presented as Δ mm [29].

### 2.8. Overt Pain-Like Behavior Evaluation

Mice were placed in clear glass compartments at room temperature. After an acclimation period of 15 min, mice were observed for 10 min, and the cumulative number of flinches was measured [27].

### 2.9. Histopathological Analysis

On the 12th day after injection of tumor cells, mice were killed, and the paws were removed and fixed in the Bowen solution (75% picric acid, 25% formaldehyde, and 5% acetic acid) for 21 days. The samples were embedded in paraffin, sectioned into 5 *μ*m sections, and stained with hematoxylin and eosin for light microscopic observation. 

### 2.10. Inactivation of the Ehrlich Tumor Cells by Thermal Alteration

The Ehrlich tumor cells were inactivated to evaluate the involvement of cellular remnants in pain induced by the Ehrlich tumor cells. For this, the cells were inactivated by the process of freezing and heating. The Ehrlich tumor cells were first suspended to the final concentration of 1 × 10^6^ or 1 × 10^7^; next cell suspension was submerged in liquid nitrogen for 5 min and then heated in water-bath (80°C) during 5 min (EvLab, Londrina, PR, Brazil). This process was repeated 5 times, followed by assessment of cell viability by the trypan blue test, in order to confirm that cells were not viable. Mice received the equivalent to 1 × 10^6^ or 1 × 10^7^ inactivated tumor cells, viable cells, or saline (25 *μ*L) i.pl. The evaluation of mechanical and thermal hyperalgesia and paw edema/tumor growth was performed between days 0 and 12 and, the evaluation of the overt pain was performed in 8th day.

### 2.11. Statistical Analysis

Results are presented as mean ± SEM of measurements made on 6 animals in each group in each experiment and are representative of two independent experiments. The two-way analysis of variance (ANOVA) was used to compare the groups and doses at all times (curves) when the hyperalgesic responses were measured at different times after the stimulus injection. The analyzed factors were treatment, time, and time *versus *treatment interaction. When there was a significant time *versus* treatment interaction, one-way ANOVA followed by Tukey's *t*-test was performed for each time. On the other hand, when the hyperalgesic responses were measured once after the stimulus injection, the differences between responses were evaluated by one-way ANOVA followed by Tukey's *t*-test. Additionally, comparative statistical analysis between two groups was done using the *t*-test. Statistical differences were considered to be significant at *P* < 0.05.

## 3. Results

### 3.1. The Subcutaneous Injection of the Ehrlich Cells Induces Mechanical and Thermal Hyperalgesia, Paw Edema/Tumor Growth, and Overt Pain-Like Behavior in a Dose-Dependent Manner

**Figure 1:**
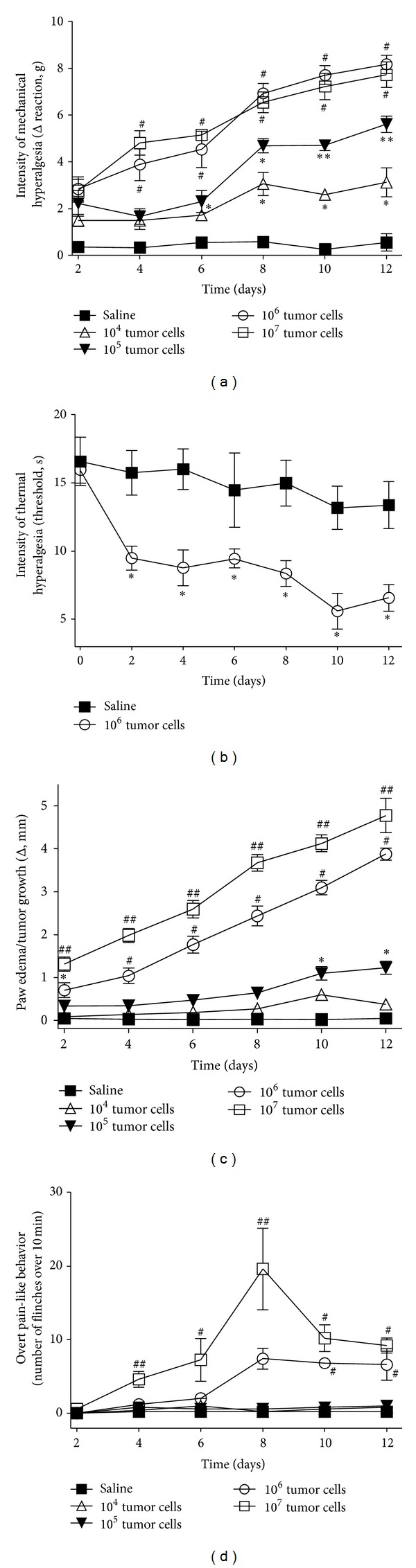
The Ehrlich tumor induces pain and paw edema/tumor growth in a dose-dependent manner. The Ehrlich tumor cells (1 × 10^4–7^) or saline (25 *μ*L) was injected subcutaneously in the paw. (a) The intensity of mechanical hyperalgesia, (b) thermal hyperalgesia, (c) paw edema/tumor growth, and (d) overt pain-like behavior was evaluated between 0 and 12 days at every-other-day intervals after injection of tumor cells or saline; *n* = 6, representative of two experiments. *P < 0.05 compared with saline; **P < 0.05 compared with saline and the dose of 10^4^; ^#^ *P* < 0.05 compared with saline and the doses of 10^4^ and 10^5^; ^##^ *P* < 0.05 compared with saline and the doses of 1 × 10^4–6^.

The Ehrlich tumor cells (1 × 10^4^–10^7^ in 25 *μ*L per paw), or the vehicle group (PBS), were subcutaneously injected in the mouse hindpaw (i.pl.), and mechanical hyperalgesia was evaluated 2, 4, 6, 8, 10, and 12 days after cell injection, Figure 1(a). The mechanical hyperalgesia induced by tumor cells was dose and time dependent. All doses of tumor cells tested induced significant mechanical hyperalgesia on day 8, which remained on days 10 and 12. There was no statistical difference between the doses of 10^6^ and 10^7^ tumor cells regarding mechanical hyperalgesia, Figure 1(a). There was no difference between the doses in the hot-plate test (data not shown); therefore, for clear presentation, only the results on thermal hyperalgesia and the dose of 10^6^ are shown in Figure 1(b). The injection of tumor cells induced a progressive and dose-dependent increase in paw edema/tumor growth, Figure 1(c), which corroborates the progressive increase of mechanical hyperalgesia in Figure 1(a). The dose of 10^4^ did not induce significant paw edema/tumor growth, while 10^5^ induced at days 10 and 12, Figure 1(c). The paw edema/tumor growth was significant between 2 and 12 days for the doses of 10^6^ and 10^7^ (Figure 1(c)). Spontaneous nociceptive behavior was quantified by the number of flinches, Figure 1(d). The doses of 10^4^ and 10^5^ did not induce paw flinch, the dose of 10^6^ induced paw flinch at days 10–12, and 10^7^ induced a significant number of flinches at days 4–12 with a peak at day 8 (Figure 1(d)). Considering these results, the dose of the 10^6^ Ehrlich tumor cells was chosen for histological analysis and behavioral experiments evaluating mechanical and thermal hyperalgesia and paw edema/tumor growth, while the dose of 10^7^ was chosen for overt pain-like behavior evaluation.

### 3.2. Histopathological Analysis

**Figure 2:**
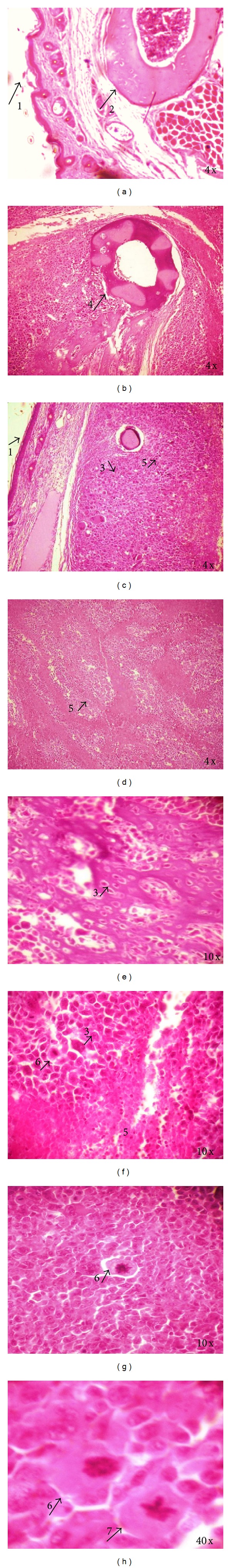
Histopathological analysis of paw injected with the Ehrlich tumor. The Ehrlich tumor cells (1 × 10^6^) or saline (25 *μ*L) was injected subcutaneously into the hindpaws of the mice. Panel (a) indicates histological sections of normal paw that received saline, and panels (b)–(h) indicate the paw that received the Ehrlich tumor cells stained by hematoxylin/eosin. Arrows (1) indicate normal epithelium; (2) normal bone cartilage; (3) tumor cells; (4) destroyed bone cartilage; (5) extensive area of necrosis; (6) tumor cells with mitosis or atypical mitosis; (7) and tumor cells with atypical nucleus.

Mice were sacrificed at day 12 after injection of the Ehrlich tumor cells or saline (25 *μ*L), and the paws were collected for histological analysis performed with hematoxylin/eosin staining (Figure 2). There was no histological abnormality in mice that received i.pl. injection of saline (Figure 2(a)), presenting normal epithelium (arrow 1) and normal bone cartilage (arrow 2). Mice that received i.pl. injection of the Ehrlich tumor cells (10^4^–10^7^) showed malignant neoplasm and poor differentiation, characterized by the presence of tumor cells, with nucleus showing frequent aberrant mitosis. Considering that there was no difference between the different doses of tumor cells regarding the tumor characteristics, the figures represent the dose of the 10^6^ Ehrlich tumor cells that was used in most of the evaluations (Figures 2(b)–2(h)).  Figure 2(b) shows bone cartilage destruction induced by tumor cells (arrow 4).  Figure 2(c) shows at 4x magnification the epithelium (arrow 1) and the presence of tumor cells (arrow 3) with intense areas of necrosis (arrow 5).  Figure 2(d) shows areas of necrosis (arrow 5) induced by the Ehrlich tumor cells.  Figure 2(e) shows at 10x magnification the presence of tumor cells (arrow 3) in a paw tissue, and Figure 2(f) shows tumor cells (arrow 3), areas of necrosis (arrow 5), and a presence of mitosis (arrow 6).  Figure 2(g) shows the presence of atypical mitosis (arrow 6), and Figure 2(h) shows at 40x magnification mitosis (arrow 6) and tumor cells with atypical nucleus (arrow 7). Therefore, the histopathological analysis confirmed the presence of the tumor cells (Figures 2(b) and 2(h)), together with an extensive area of necrosis (Figures 2(c), 2(d), and 2(f)) characterized by neutrophilic infiltration, associated with the presence of fibrin and red blood cells, which gives an eosinophilic coloration (Figure 2(d)), tumor cells with aberrant mitosis (Figure 2(g)), and bone/cartilage destruction (Figure 2(b)).

### 3.3. Inactivation of the Ehrlich Tumor Cells by Thermal Alteration Abolishes the Nociceptive Responses

**Figure 3:**
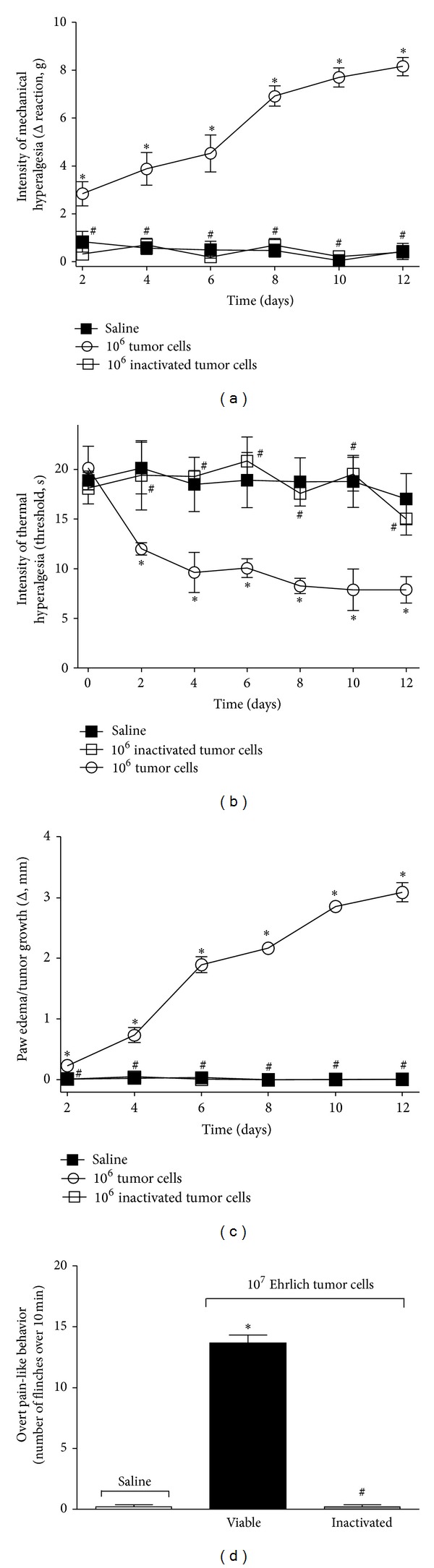
Thermal inactivation of the Ehrlich tumor cells abolishes pain and paw edema/tumor growth. The Ehrlich tumor cells (1 × 10^6^) were inactivated by cold followed by heat. Mice received 25 *μ*L of inactivated tumor cells, viable tumor cells (1 × 10^6^), or saline. (a) The intensity of mechanical hyperalgesia, (b) thermal hyperalgesia, and (c) paw edema/tumor growth was evaluated on days 2–12 after injection of the inactivated Ehrlich tumor cells, viable tumor cells, or saline. Mice received 1 × 10^7^of inactivated tumor cells, viable cells, or saline and (d) the overt pain was evaluated on the 8th day after injection; *n* = 6, representative of two experiments. *P < 0.05 compared with the saline, and ^#^ *P* < 0.05 compared with the viable 1 × 10^6^ or 1 × 10^7^ tumor cells.

Mice received i.pl. injection of saline (25 *μ*L), the viable Ehrlich tumor cells (10^6^ or 10^7^/paw), or the inactivated Ehrlich tumor cells (equivalent to 10^6^ or 10^7^ cells). Mechanical and thermal hyperalgesia and paw edema/tumor growth were evaluated between 0 and 12 days, and overt pain-like behavior was evaluated on day 8 after stimulus. The inactivation of the 10^6^ Ehrlich tumor cells was able to abolish the mechanical (Figure 3(a)) and thermal (Figure 3(b)) hyperalgesia and paw edema/tumor growth (Figure 3(c)) compared with the viable cells. Inactivation of the 10^7^ tumor cells also resulted in abolishment of overt pain-like behavior (Figure 3(d)) compared with the viable cells. Thus, the cellular remnants of the Ehrlich tumor cells were not capable of inducing paw edema/tumor growth and nociceptive responses, which suggests that these responses depend on the proliferation of tumor cells and their activities and interactions with the host immune responses.

### 3.4. Effect of Indomethacin Treatment on the Nociceptive Responses and Paw Edema/Tumor Growth Induced by the Ehrlich Tumor Cells

**Figure 4:**
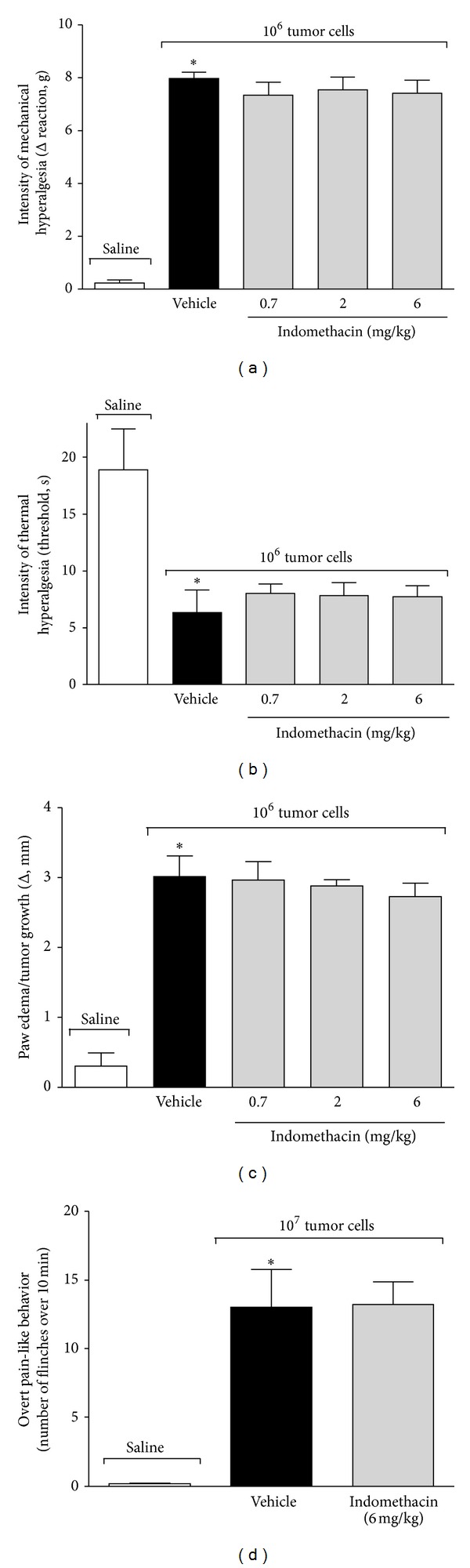
Effect of indomethacin treatment on pain and paw edema/tumor growth induced by the Ehrlich tumor cells. Mice received the 1 × 10^6^ Ehrlich tumor cells or saline, and on the 8th day, they were treated with indomethacin (0.7–6 mg/kg, i.p.) or Tris buffer. (a) The intensity of mechanical hyperalgesia, (b) thermal hyperalgesia, and (c) paw edema/tumor growth was evaluated 3 h after the treatment. Mice received the 1 × 10^7^ Ehrlich tumor cells or saline, and on 8th day, they were treated with indomethacin (6 mg/kg i.p.) or Tris buffer, and (d) overt pain was assessed 3 h after treatment; *n* = 6, representative of two experiments. *P < 0.05 vehicle group compared with the saline.

Mice received the 10^6^ or 10^7^ Ehrlich tumor cells, and on the 8th day, they were treated with indomethacin (0.7, 2, or 6 mg/Kg i.p.) or Tris buffer, and 3 h after the treatment mechanical, and thermal hyperalgesia, paw edema/tumor growth, and overt pain-like behavior were measured (Figure 4). The Ehrlich tumor cells induced significant mechanical (Figure 4(a)) and thermal (Figure 4(b)) hyperalgesia, paw edema/tumor growth (Figure 4(c)), and overt pain-like behavior (Figure 4(d)) compared with the saline group. However, the treatment with indomethacin did not affect those parameters induced by the Ehrlich tumor cells (Figure 4), indicating that they do not depend on the production of prostanoids.

### 3.5. Effect of Amitriptyline Treatment on the Nociceptive Responses and Paw Edema/Tumor Growth Induced by the Ehrlich Tumor Cells

**Figure 5:**
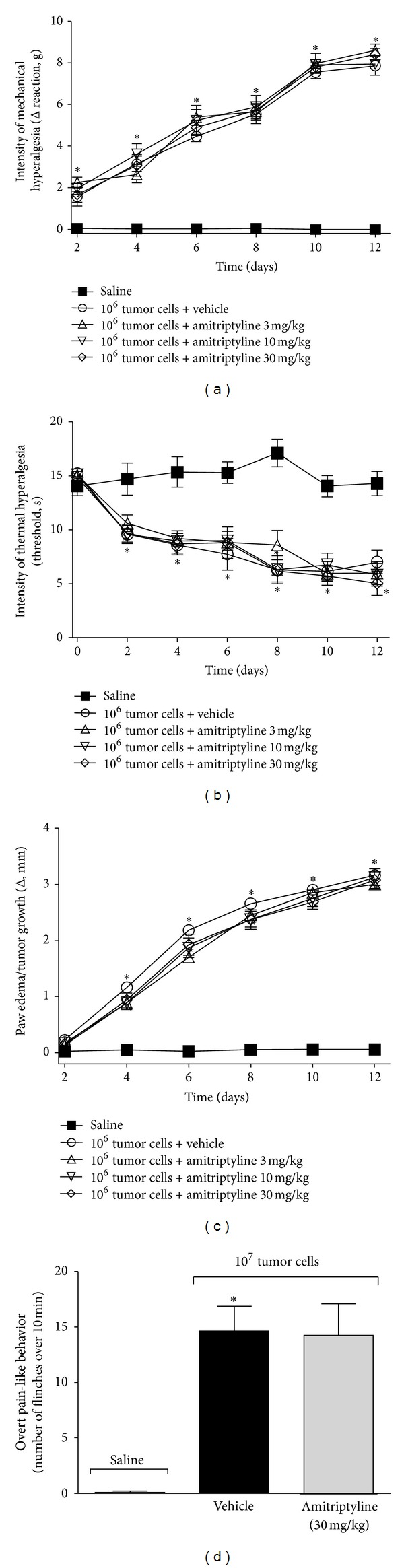
Effect of amitriptyline treatment on pain and paw edema/tumor growth induced by the Ehrlich tumor cells. Mice received the 1 × 10^6^ Ehrlich tumor cells or saline, and they were treated with amitriptyline (3–30 mg/kg, p.o.) or water every day after subcutaneous injection of tumor cells. (a) The intensity of mechanical hyperalgesia, (b) thermal hyperalgesia, and (c) paw edema/tumor growth was evaluated 3 h after the treatment on days 2, 4, 6, 8, 10, and 12 after injection of the cells. Mice received the 1 × 10^7^ Ehrlich tumor cells or saline and were treated daily with amitriptyline (30 mg/kg, p.o.) or water and after 8 days; (d) the overt pain was assessed 3 h after the treatment; *n* = 6, representative of two experiments. *P < 0.05 compared with the saline.

After inoculation of the 10^6^ or 10^7^ Ehrlich tumor cells, mice were treated with amitriptyline (3, 10, and 30 mg/kg) or water via oral gavage (per oral: p.o.) once a day during 12 days, and 3 h after treatment, mechanical and thermal hyperalgesia, paw edema/tumor growth, and overt pain-like behavior were evaluated (Figure 5). None of the doses of amitriptyline affected the Ehrlich tumor cells-induced mechanical hyperalgesia (Figure 5(a)), thermal hyperalgesia (Figure 5(b)), paw edema/tumor growth (Figure 5(c)), or overt pain (Figure 5(d)). These results suggest that the inhibition of serotonin and/or norepinephrine reuptake does not affect the maintenance of cancer pain in this model.

### 3.6. Effect of Morphine Treatment on the Nociceptive Responses and Paw Edema/Tumor Growth Induced by the Ehrlich Tumor Cells

**Figure 6:**
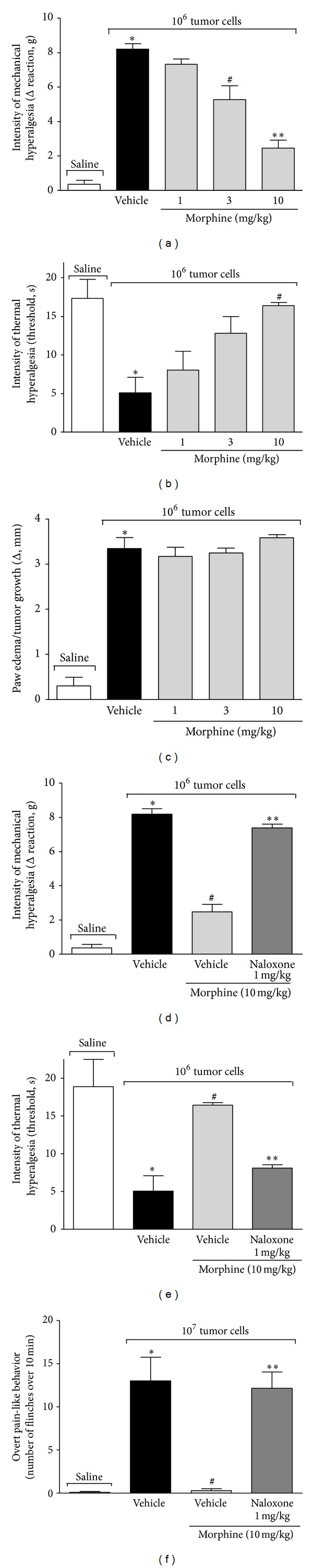
Effect of morphine treatment on pain and paw edema/tumor growth induced by the Ehrlich tumor cells. Mice that received the 1 × 10^6^ Ehrlich tumor cells were treated with morphine (1–10 mg/Kg, i.p.) or saline on the 8th day after tumor cells injection. (a) The intensity of mechanical hyperalgesia, (b) thermal hyperalgesia, and (c) paw edema/tumor growth was evaluated 45 minutes after treatment with morphine. In another set, mice were treated with naloxone (1 mg/kg, i.p.) 1 hour before the treatment with morphine (10 mg/kg i.p.), and (d) 45 minutes after morphine treatment, the intensity of mechanical hyperalgesia and (e) thermal hyperalgesia was evaluated. Mice received the 1 × 10^7^ Ehrlich tumor cells or saline, and after 8 days, they were treated with naloxone (1 mg/kg i.p.) 1 h before treatment with morphine (10 mg/kg i.p.), and (f) 45 min after the treatment with morphine, the overt pain was assessed; *n* = 6, representative of two experiments. *P < 0.05 vehicle group compared with the saline; ^#^ *P* < 0.05 compared with the tumor or compared with the treatment with naloxone plus morphine, and **P < 0.05 compared with the doses of 1 and 3 mg/kg morphine.

Mice were treated with morphine (1–10 mg/kg, i.p.) or saline on the 8th day after the Ehrlich tumor (10^6^ or 10^7^ cells) injection in which the peak of hyperalgesia was detected. After the treatment (45 min) with morphine, mechanical (Figure 6(a)) and thermal (Figure 6(b)) hyperalgesia and paw edema/tumor growth (Figure 6(c)) were evaluated. The morphine dose dependently inhibited Ehrlich tumor-induced mechanical (Figure 6(a)) and thermal (Figure 6(b)) hyperalgesia, but it did not affect the paw edema/tumor growth, which indicates that morphine presents analgesic effect not related to inhibition of tumor proliferation. The dose of 3 mg/kg of morphine reduced the Ehrlich tumor-induced mechanical hyperalgesia compared with the positive control, while the dose of 10 mg/kg of morphine presented significant inhibition compared with the Ehrlich tumor-positive control and the doses of 1 and 3 mg/kg of morphine (Figure 6(a)). The Ehrlich tumor-induced thermal hyperalgesia was inhibited by the dose of 10 mg/kg of morphine without significant inhibition with the doses of 1 and 3 mg/kg (Figure 6(b)). To confirm the receptor-dependent effect of morphine and that an opioid-receptor-dependent inhibition of the Ehrlich tumor-induced hyperalgesia was being observed, mice were treated with naloxone (1 mg/kg, i.p.) 1 h before morphine (10 mg/kg) treatment, and after additional 45 min, measurements of mechanical (Figure 6(d)) and thermal (Figure 6(e)) hyperalgesia were performed. Again, the Ehrlich tumor-induced (10^6^ cells) mechanical and thermal hyperalgesia were inhibited by morphine, and this inhibition was prevented by naloxone treatment. Furthermore, the Ehrlich tumor induced (10^7^ cells) spontaneous flinches at the 8th day of cancer development, which were also inhibited by morphine treatment (10 mg/kg), and the analgesic effect of morphine was prevented by naloxone treatment (Figure 6(f)). Therefore, the Ehrlich tumor induces mechanical and thermal hyperalgesia and overt pain-like behavior susceptible to opioid-receptor analgesia (Figure 6).

## 4. Discussion

Cancer pain directly affects the quality of life and survival of patients with cancer [11, 30]. Cancer pain is characterized by the presence of hyperalgesia, allodynia, and/or spontaneous pain. Tactile allodynia and mechanical hyperalgesia are important features of cancer pain and decrease the life quality of patients. Considering the importance of pain in cancer, several experimental models, including neuropathic cancer pain [11], bone cancer pain [12, 31, 32], and cancer pain induced by orthotopic tumor inoculation in mice [10, 33], have been developed and contributed to the characterization of the pathophysiology of cancer pain.

Several experimental studies have shown that marked nociceptive reactions induced by malignant tumor vary with animal species, tumor types, and localizations of the tumor [10–12, 33–36]. In the present study, we develop a model of pain characterized by mechanical and thermal hyperalgesia and spontaneous pain-like behavior, for example, flinching of the paw. The mechanical hyperalgesia and flinches were dependent on the number of the Ehrlich tumor cells injection and were progressive over time. The time- and dose-dependent features of the present model argue in its favor as a good model to investigate the effect of novel analgesics and mechanisms involved in cancer pain regarding mechanical hyperalgesia and overt pain-like behavior. It is noteworthy that, in the case of thermal hyperalgesia, it was significant and increased over time, but there were no differences in the responses induced by different number of the Ehrlich tumor cells injected.

It is important to understand the mechanisms involved in the model used to investigate the action of novel drugs and to have a clear view of the possible mechanisms to be addressed. Nevertheless, as a first insight into the mechanisms involved in the Ehrlich tumor-induced nociception, it was determined its susceptibility to three classes of analgesics; nonsteroidal anti-inflammatory drug, tricyclic antidepressant, and opioid. The acute treatment with nonsteroidal anti-inflammatory drug, indomethacin, a cyclooxygenase inhibitor, did not affect the nociceptive responses and paw edema/tumor growth induced by the Ehrlich tumor. Indomethacin did not affect pain in a model of femur cancer pain induced by fibrosarcoma cells in mice [37]. On the other hand, in a model of bone cancer pain induced by injection of osteolytic murine sarcoma into the femur, the oral administration of indomethacin reduced pain behavior in mice [38]. These controversial data may be due to the different routes of administration that were used, the different doses of treatment, and mainly the different models of cancer pain.

Chronic treatment with amitriptyline, a tricyclic antidepressant inhibitor of reuptake of serotonin and norepinephrine, did not inhibit the nociception induced by the Ehrlich tumor cells. Others have shown that amitriptyline reduced only spontaneous pain behavior at sedative doses [37]. Tricyclic antidepressants have been extensively studied because there is evidence of their analgesic properties in several chronic diseases [37], and neuropathic pain [39]. However, the reuptake of serotonin and norepinephrine seems not to be related to the maintenance of cancer pain induced by the Ehrlich tumor. 

The treatment with morphine dose dependently reduced the nociception induced by the Ehrlich tumor. Additionally, it was observed that the effect of morphine was receptor specific, because the opioid receptor antagonist naloxone reversed the effect of morphine. Despite the reduction of nociception promoted by morphine, there was no change in tumor growth, which indicates that morphine inhibited nociceptive responses rather than reduced nociception by decreasing tumor growth. 

Despite all of the research performed in an attempt to inhibit cancer pain, it cannot be stated the exact mechanisms involved in the maintenance and chronicity of cancer pain. In fact, cancer patients still face inadequate analgesia. One major reason is that, despite some similarities, each model of cancer pain has its peculiar mechanisms similarly to each type of cancer in humans. Thus, it is conceivable that a great variety of cancer pain models are necessary to line up with varied human conditions. Bone cancer pain models are considered particularly interesting since during metastasis tumor cells may reach the bones. In the present study, there was bone cartilage destruction in the foci of tumor injected, indicating that there might be a bone pain component in this model. Nevertheless, models that evaluate the pain before metastasis are also important. To exemplify conditions in which cancer pain before metastasis is important, it is noteworthy to mention that a third of breast cancer patients will report pain in the lump spontaneously or upon examination [40]. The present model using the injection of cells of a murine mammary adenocarcinoma presents a condition resembling the preoperative breast cancer pain since there is spontaneous pain-like behavior in the paw and hyperalgesia upon stimulation of the lump (foci of tumor injection in the paw). Importantly, there is a significant relation between preoperative breast pain and phantom breast pain syndrome [41], and treatment of pain prior to mastectomy is an important clinical approach to reduce the incidence of phantom breast pain syndrome. Therefore, the present model might contribute as a model to study preoperative breast cancer pain mechanisms.

## 5. Conclusion

We have characterized a cancer pain model induced by subcutaneous injection of the Ehrlich tumor cells into the hindpaw of mice. This model is characterized by robust tumor growth and rapid development of mechanical and thermal hyperalgesia and overt pain-like behavior, rendering it as convenient to study the mechanisms of cancer pain and tumor growth and to test new treatments.
